# Association between Mandibulofacial Asymmetry and Temporomandibular Disorder Using Diagnostic Criteria for Temporomandibular Disorder (DC/TMD)

**DOI:** 10.1055/s-0045-1809698

**Published:** 2025-08-05

**Authors:** Agia T. Andriani, Maria Purbiati, Krisnawati, Ira Tanti

**Affiliations:** 1 Orthodontic Residency Program, Faculty of Dentistry, Universitas Indonesia, Jakarta, Indonesia; 2 Department of Orthodontics, Faculty of Dentistry, Universitas Indonesia, Jakarta, Indonesia; 3 Department of Prosthodontics, Faculty of Dentistry, Universitas Indonesia, Jakarta, Indonesia

**Keywords:** facial asymmetry, temporomandibular joint disorders, photography, diagnostic techniques and procedures, diagnosis

## Abstract

**Objective:**

Mandibulofacial asymmetry can cause disharmony in facial appearance and smile and may also impair stomatognathic function and speech ability, negatively affecting psychological well-being and quality of life. This condition is also commonly associated with temporomandibular disorders (TMDs). This study aimed to analyze the relationship between mandibulofacial asymmetry and TMDs.

**Materials and Methods:**

This study conducted a cross-sectional study involving 42 patients (14 males and 28 females) aged 17 to 45 years. The diagnosis of TMDs was made using the Diagnostic Criteria for Temporomandibular Disorders, and facial asymmetry was assessed using frontal photography.

**Result:**

The results showed that 76.2% of the patients with mandibulofacial asymmetry had a TMD. Statistical tests showed a relationship between mandibulofacial asymmetry and TMDs ( *p* -value < 0.05).

**Conclusion:**

The TMD is closely related to mandibulofacial asymmetry. However, the relationship between mandibulofacial asymmetry and the specific categories of TMDs was not statistically significant, as determined by multinomial regression analysis.

## Introduction

Mandibulofacial asymmetry is characterized by an imbalance or disproportion in the facial features, particularly affecting the mandible and chin. This asymmetry can result in visible disharmony in facial appearance and an uneven smile, which may lead to significant aesthetic concerns for affected individuals. Beyond the cosmetic implications, mandibulofacial asymmetry can also have functional consequences, particularly in the stomatognathic system, which includes the structures involved in chewing, speech, and swallowing. Impaired function in these areas can contribute to difficulties in speech articulation and masticatory efficiency, ultimately impacting an individual's psychological well-being and overall quality of life. 1

The temporomandibular joint (TMJ) is a bilateral structure; it will cause trauma if there is a change in balance between the two sides. The TMJ disorder (TMD) in the jaw joint involves muscles, mastication, and surrounding tissue structures. 2 Studies by Choi et al have indicated a correlation between TMDs accompanied by joint disc displacement and the direction of chin deviation. Specifically, chin deviation tends to align with the side afflicted by TMD. 3

TMD can present with various symptoms, including joint pain, muscle tenderness, limited jaw movement, and audible clicking or popping sounds in the joint. The relationship between mandibulofacial asymmetry and TMD has been the subject of numerous studies, with evidence suggesting that the asymmetry may contribute to the development or exacerbation of TMD symptoms. 1 These symptoms are frequently observed in individuals with class III skeletal malocclusion characterized by mandibular prognathism, often accompanied by facial asymmetry. Tervahauta et al, in their study, showed that there is a relationship between canine relations and TMDs, where class II occlusal relations are also associated with TMDs. 4 Posterior crossbite is a hallmark feature of mandibulofacial asymmetry, typically manifested by a misalignment of one or more posterior teeth. Maglione et al found an association between facial and/or condyle asymmetry and TMJ internal derangement. 5 The relationship between unilateral and bilateral posterior crossbites and TMDs remains controversial. 6 In their study, Khayat et al found a correlation between posterior crossbites and symptoms of TMDs, especially arthralgia, myalgia, and pain in the TMJ. 7 TMDs can be pain related (pain-related TMD and headache), disorders involving the joints (intra-articular and degenerative joint disorders), or a combination of pain and joint disorders. 8

Cephalometric radiography can provide a picture of skeletal asymmetry, but the perception of mandibulofacial asymmetry is also determined based on the state of the face's soft tissues. The state of the soft tissue structure dramatically influences the patient's decision to perform treatment and the satisfaction of treatment outcomes. 9 Soft tissue morphology is highly correlated with the underlying skeletal structures. 10 Examination using digital photography can provide an overview of the patient's mandibulofacial soft tissue to determine treatment plans, evaluate treatment outcomes, and communicate with patients. 11

The TMD examination index is handy in studying the incidence of TMDs in specific populations, tracing etiological factors, and measuring the effectiveness of a given therapy. Several measurement indices for TMDs have been developed. 13 14 15 The Diagnostic Criteria for Temporomandibular Disorder (DC/TMD) provides a standardized framework for diagnosing TMDs, and it is divided into two main sections: clinical diagnosis and psychosocial assessment. In Axis I: Clinical Diagnosis, TMD is classified into three main groups based on physical symptoms. Group I: muscle disorders (myalgia) and disc displacement disorders cover cases where the disc in the TMJ becomes displaced, and joint disorders involve conditions affecting the TMJ, such as arthralgia, which is characterized by joint pain and osteoarthritis or osteoarthrosis, where there is structural degeneration of the joint, often accompanied by joint sounds like crepitus. Axis II: Psychosocial Assessment focuses on how TMD affects a patient's quality of life, particularly in terms of pain-related disability and psychological distress. 16

This study aims to analyze the correlation between mandibulofacial asymmetry and TMDs using the DC/TMD for diagnosis and frontal photography for facial asymmetry assessment.

## Materials and Methods

This study is a cross-sectional analytical observational study that has obtained a Certificate of Passing Ethics from the Research Ethics Commission of the Faculty of Dentistry, University of Indonesia with number: 67/ *Ethical Approval* /FKGUI/IX/2022, and the Ethics Commission of RSKGM FKG UI with number: 059/UN2. F2. ORTO/P *p* .00.00/2022. Data was obtained from 42 respondents as research subjects.

Inclusion criteria included respondents aged 17 to 45 years with fully erupted permanent teeth (up to the second molars) and either mandibulofacial asymmetry (menton deviation > 3 mm) or symmetry (menton deviation < 3 mm). Exclusion criteria included respondents with a history of orthodontic treatment, orthognathic surgery, neuromuscular disorders, facial trauma, or habits such as bruxism or unilateral chewing.

**Fig. 1:**
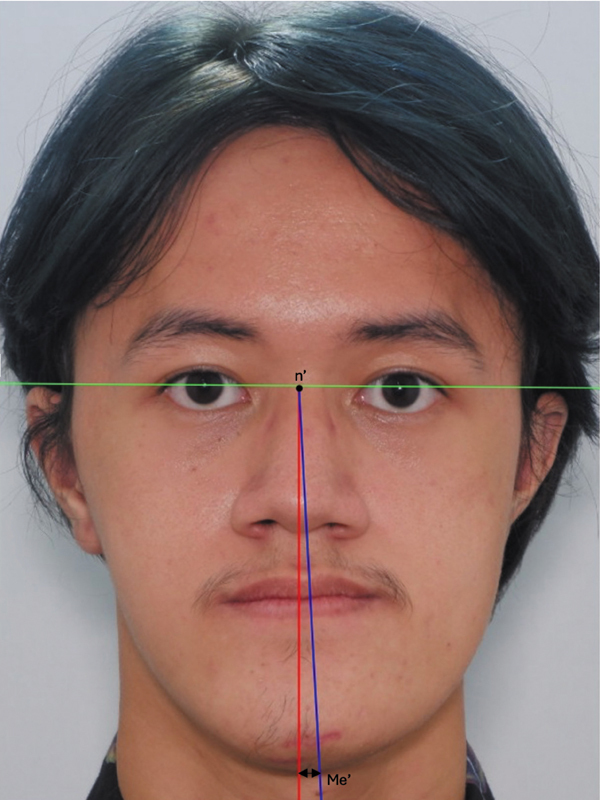
Extraoral photographic examination to determine facial asymmetry, i.e., chin deviation, by measuring the N' – Me' point deviation with reference line are the interpupillary line (green line) and facial midline (redline) perpendicular to the interpupillary line. The subject includes an asymmetric category when there is a deviation of the Me' point to the facial midline point > 3 mm. 19

All photographs were taken by the same experienced photographer based on the standards of the European Association for Cranio-Maxillo-Facial Surgery. Photographs (resolution 2496 × 1664 pixels) were taken with a Fujifilm X-A5 digital camera (Fujifilm Corp., Tokyo, Japan). Indirect anthropometric measurements for facial asymmetry examination are determined based on measurements of the patient's face photos using measuring instruments in digital photographs using CorelDraw (Corel Corp., Ottawa, Canada) by an experienced observer blinded to patient information. Fig. 1 explains an extraoral photographic examination to determine facial asymmetry by measuring the N' – Me' point deviation with reference lines: the interpupillary line (green line) and facial midline (line) perpendicular to the interpupillary line. The subject includes an asymmetric category when there is a deviation of the Me' point to the facial midline point > 3 mm. 19

The diagnostic process begins with history taking, where the patient's medical and symptom history is collected, followed by a physical examination using the DC/TMD axis I questionnaire. The symptom questionnaire is filled out using the guided question method, while the DC/TMD axis I questionnaire is performed using physical examinations. Physical examinations include palpation of muscles and joints, observing jaw movements for deviations or limitations, and sounds in the joints like clicking or crepitus. The results from the questionnaire are then entered into a diagnostic decision-making chart. 20

## Research Results

The reliability of measurement results is obtained by conducting interobserver and intraobserver tests using the intraclass correlation (ICC) test. Interobserver and intraobserver tests are performed by measuring asymmetry deviations in each group. Based on the ICC test (> 0.80), there is excellent agreement on the interobserver and intraobserver tests.

Based on the results obtained, it is known that the nonasymmetry and asymmetry groups have the same large presentation. Table 1 shows that the majority of subjects with TMDs suffered from joint disorders (26.2%), followed by the group suffering from pain disorders (23.8%). The group with pain and joint disorders had the smallest presentation (7.1%). The group of subjects without TMDs had the most significant percentage, at 42.9%.

**Table 1:** Distribution of nonasymmetry, asymmetry, and TMD subject data

Variable	*n*	Frequency
Facial symmetry		
- Nonasymmetry	21	50%
- Asymmetry	21	50%
TMD		
- No distractions	18	42.9%
- Pain disorders	10	2.8%
- Joint disorders	11	26.2%
- Pain and joint disorders	3	7.1%

Abbreviation: TMD, temporomandibular disorder.

In Table 2 , nonasymmetry and asymmetry subjects who experienced joint disorders had a balanced percentage, but no subjects experienced joint disorders and pain. The subject of asymmetry with joint disorders is the most commonly found disorder, followed by pain disorders and both pain and joint disorders. Table 2 shows that in the nonasymmetry group, most subjects did not have TMDs compared with those in the asymmetry group who did not have TMDs. A chi-square test showed the relationship between facial asymmetry and TMDs. Further analysis showed that in the asymmetry group, TMDs occurred with a percentage of 76.2%. Further statistical test results in a *p* -value of 0.029 ( *p* -value < 0.05). Therefore, hypothesis 1 was accepted; there is a significant relationship between facial asymmetry and TMDs.

**Table 2:** Chi-square test between facial symmetry and overall temporomandibular joint disorder

Symmetry	Non-TMD		TMD		Total, *n* (%)	*p* -Value
	*n*	%	*n*	%		
Nonasymmetry	13	61.9	8	38.1	21 (100)	0.029 a
**Asymmetry**	5	23.8	16	76.2	21 (100)	

Abbreviation: TMD, temporomandibular disorder.

a Significant if *p* -value < 0.05.

In Table 3 , multinomial regression analysis revealed no significant relationship between facial asymmetry and specific TMD categories, such as pain disorders, joint disorders, or a combination of both. Further analysis using a multinomial regression test with a baseline of no disorders showed that there was no association between facial asymmetry and TMDs with pain disorders, joint disorders, and a combination of both disorders ( *p* -value > 0.05), no disorders 0 (see Table 3 ). Therefore, hypotheses 2, 3, and 4 were rejected. Namely, there is no relationship between facial asymmetry and TMDs of the pain disorder group, joint disorder group, and combined group of disorders of pain and joints.

**Table 3:** Multinomial regression test for asymmetry with temporomandibular joint disorders

	None			Pain disorders			Joint disorders			Pain and joint disorders		
	*n*	%	*p* -Value	*n*	%	*p* -Value	*n*	%	*p* -Value	*n*	%	*p* -Value
Nonasymmetry	13	61.9	** 1 a**	4	19.0	**0.102**	4	19.0	**0.064**	0	0	**0**
**Asymmetry**	5	23.8		6	28.6		7	33.3		3	14.3	
Total	18	42.9		10	23.8		11	26.2		3	14.3	

a Baseline = No interference.

In Table 4 , the chi-square test shows the relationship between the side with TMD and the side with mandibular deviation subjects with more left mandibular deviation (76.1%) than right mandibular (23.9%). Five (31.2%) subjects with a left mandibular deviation did not have TMDs; there were four (25%) subjects with right-sided TMDs and four (25%) subjects with left-sided TMDs. In comparison, there were three (18.8%) subjects who experienced TMDs on both sides. Two (40%) subjects with a right mandibular deviation did not have TMDs, while there were two (40%) subjects with left-sided TMDs and one (20%) subjects who had TMDs on both the right and left sides. There were no subjects who had TMDs on the right side. Further analysis tests yielded a *p* -value of 0.650, thus showing no significant relationship between the side with TMDs and the side with mandibular deviation ( *p* -value < 0.05), so hypothesis 5 was rejected.

**Table 4:** Chi-square test between the side with temporomandibular joint disorder and the side with mandibular deviation

TMD	Midline deviation		Total	*p* -Value
	Right	Left	*n* (%)	
	*n* (%)	*n* (%)		
No TMD	2 (28.6)	5 (71.4)	7 (100)	0.650
Right	0 (00.0)	4 (100.0)	4 (100)	
Left	2 (33.3)	4 (66.7)	6 (100)	
Right and left	1 (25.0)	3 (75.0)	4 (100)	

Abbreviation: TMD, temporomandibular disorder.

a Significant if *p* -value < 0.05.

## Discussion

The TMD is one of the causes of pain in the orofacial area and can cause persistent or chronic pain. Chronic disorders of the joints can be associated with other chronic pain conditions, including migraines, fibromyalgia, and the spread of pain in the orofacial area. Proper diagnosis and treatment are vital in obtaining a good prognosis and reducing the effects of TMD on patients' quality of life. 21 The proportion of facial soft tissues can be measured using photos of the frontal direction of the face. Edler et al, in their research, revealed that there is a relationship between frontal facial photographs and measurements on cephalometric radiographs. 22 Haraguchi et al obtained the deviation of the menton point on the examination of the frontal face photo, which is related to the deviation of the menton point on posteroanterior cephalometric examination. The midline of the face is the perpendicular line between the left and right pupils, while the menton (Me') point of soft tissue is the lowest point of the outer contour of the face. 23

This study was a cross-sectional, analytical, observational study. Data was collected once on respondents who had met the inclusion and exclusion criteria through guided interviews, clinical examinations using DC/TMD questionnaires, and facial photos of frontal direction performed by one operator. In this study, the evaluation of facial asymmetry using frontal facial images was performed on 42 subjects consisting of 21 nonasymmetry subjects and 21 asymmetry subjects. The female subject group had a more extensive presentation of 66.6% compared with males. In their study, Djordjevic et al found no significant difference between symmetry in the lower third of the face and sex. 24 The age range of subjects taken was 17 to 45 years. The mean age of the asymmetry subject was 27.43 ± 6.183 years, while that of the nonasymmetry subject was 25.86 ± 6.145 years. Ferrario et al, in their research, found no significant relationship between age and sex with facial asymmetry; the study was conducted on subjects aged 12 to 56 years. 25

For the assessment of mandibular joint disorders, in this study, it was found that most subjects had TMDs, both in muscles and joints and a combination of pain or functional impairment. The result showed most subjects who came for orthodontic treatment had TMDs after examination using the DC/TMD diagnostic index, which was 57.1% of the total subjects. This finding aligns with the research of Iturriaga et al, which states that 50% of the population has TMDs. 26

Facial asymmetry is associated with TMDs due to changes in the shape of the condyle head due to TMDs. Changes in the shape of the condyle head can cause disturbances in the joints. This study showed that in the asymmetry group, there was a TMD in 76.2% of asymmetry subjects. Further statistical tests showed a relationship between facial asymmetry and TMDs ( *p* -value < 0.05). In their study, Noh and Lee found that mandibular asymmetry can increase the risk of TMDs. 26 27

TMDs can be caused by various factors: occlusion, trauma, parafunctional, and psychological disorders. In the state of facial asymmetry, there is a balance disturbance between the two sides of the face. The TMJ is a bilateral structure; if there is interference on one side, microtrauma will occur. In their study, Choi et al found that subjects with more than 3 mm facial asymmetry had a greater risk of TMDs. 3 Contrary to the results of previous studies, Luz et al argued that there was no relationship between mandibular asymmetry and TMDs. This research shows a significant association between facial asymmetry and TMDs. In contrast, multinomial regression analysis did not reveal a statistically significant relationship between facial asymmetry and specific TMD categories. The absence of a significant association may reflect the multifactorial nature of TMD, where occlusion, trauma, parafunctional habits, and psychological factors all play a role. 28

In their research, Choi et al found TMDs accompanied by a relation to the direction of chin deviation, where the direction of chin deviation is the same as the side experiencing TMDs. Contrary to previous studies, the results of the current study show no significant relationship between the side with TMDs and the side with a healthy side experiencing mandibular deviation ( *p* -Value > 0.05). 3 29 30

The research has limitations that affect the generalizability and depth of its findings. The study involved a relatively small sample size of 42 patients (14 males and 28 females) aged 17 to 45 years. This limited sample may not accurately represent the wider population, potentially restricting the ability to apply the results to other age groups or individuals from different demographic backgrounds. A more extensive and more diverse sample could provide more comprehensive insights. These limitations suggest that further research with a larger, more diverse sample and improved assessment techniques is needed to validate and expand upon the findings.

## Conclusion

The findings of this study indicate a significant association between facial asymmetry and TMDs, suggesting that structural imbalances in the mandible may contribute to the development of TMD. However, the relationship between facial asymmetry and specific categories of TMDs—such as pain-related conditions or joint disorders—was not found to be significant. These results are consistent with existing literature highlighting the complex interplay between mandibulofacial asymmetry and TMD. Future research with larger, more diverse samples is needed to better understand the clinical implications and improve diagnostic and therapeutic strategies.
